# Efficacy of Touch Imprint Cytology in Intraoperative Diagnosis of Invasive Mucinous Adenocarcinoma of the Lung: A Case Report and Literature Review

**DOI:** 10.3390/clinpract14010019

**Published:** 2024-01-29

**Authors:** Toshihiko Kato, Yumiko Higuchi, Mei Oshima, Fuki Endo, Fuminori Sato, Shiro Sugihara, Manabu Yamamoto, Yasuo Imai

**Affiliations:** 1 Department of Diagnostic Pathology, Ota Memorial Hospital, SUBARU Health Insurance Society, 455-1 Oshima, Ota City, Gunma 373-8585, Japan; mt.katou3371@gmail.com (T.K.); leo3326272@gmail.com (Y.H.); yayi49257@gmail.com (M.O.); smygu85067@yahoo.co.jp (F.E.); korosuke359@gmail.com (F.S.); ota_hp_byouri@yahoo.co.jp (S.S.); 2 Department of General Thoracic Surgery, Ota Memorial Hospital, SUBARU Health Insurance Society, 455-1 Oshima, Ota City, Gunma 373-8585, Japan; manabuyamamoto1110@gmail.com

**Keywords:** invasive mucinous adenocarcinoma, frozen section diagnosis, touch imprint cytology, nuclear inclusion, mucus

## Abstract

A preoperative diagnosis of the peripheral small lung nodule is often difficult, and an intraoperative frozen section diagnosis (FSD) is performed to guide treatment strategy. However, invasive mucinous adenocarcinoma (IMA) is prone to be overlooked because of the low sample quality and weak atypia. We herein report a case of IMA, in which touch imprint cytology (TIC) revealed diagnostic efficacy. A 74-year-old male with a small, subsolid nodule in the right upper lobe underwent a thoracoscopic wedge resection. A grayish brown, 10 × 7 mm-sized nodule was observed on the cut surface. Intraoperative FSD revealed lung tissue with mild alveolar septal thickening and stromal fibrosis but without overt atypia. Meanwhile, TIC revealed mucus and a few epithelial cells with intranuclear inclusions, which pathologists evaluated as reactive. Finally, focal organizing pneumonia was tentatively diagnosed, and surgery was finished without any additional resection. However, permanent section diagnosis revealed a microinvasive mucinous adenocarcinoma. Nuclear inclusions were confirmed in tumor cells. In the intraoperative setting, TIC may be more advantageous than FSD in observing nuclear inclusions and mucus. Mucinous background and nuclear inclusion on TIC may suggest IMA even if FSD does not suggest malignancy in an intraoperative diagnosis of the peripheral small lung nodule.

## 1. Introduction

Invasive mucinous adenocarcinoma (IMA) (referred to as mucinous bronchioloalveolar carcinoma (BAC) in the 2004 World Health Organization (WHO) classification [1]) is a unique subtype of adenocarcinoma, consisting of tumor cells showing the morphology of goblet cells or tall columnar cells, containing abundant intracytoplasmic mucus [2]. IMA accounts for approximately 2–10% of lung adenocarcinoma [3,4,5]. According to the analysis of primary lung cancer between 2000 and 2014 extracted from the Surveillance, Epidemiology, and End Results (SEER) database, IMA is more frequent in females than in males (male: female ratio 1:1.45), while male: female ratio is 1:1.09 in invasive non-mucinous adenocarcinoma (INMA) [6]. The average age at diagnosis of IMA patients was similar to that of patients with INMA (65.97 vs. 66.09 years old) [6]. The association of IMA with smoking is presently controversial [7], but smoking may have an unfavorable effect on the prognosis [8]. IMA, generally considered to be non-terminal respiratory unit (TRU) type-adenocarcinoma, typically expresses cytokeratin (CK) 7 (88–94.7%) and CK20 (~79%) but rarely expresses a thyroid transcription factor (TTF)-1 (11–27.5%) and novel aspartic proteinase of the pepsin family A (napsin A) (11%) [7]. Non-TRU type tumors are associated with a worse prognosis compared with TRU-type tumors, 88% of which expressed TTF-1 [7]. IMA has characteristic molecular features, such as frequent Kirsten rat sarcoma viral oncogene homolog (KRAS) mutations (63–90%), neuregulin-1 (NRG1) fusion (7–27%), and rare epidermal growth factor receptor (EGFR) mutations (0–5%), thus representing substantial genetic differences from INMA [7]. Anaplastic lymphoma kinase (ALK) rearrangements have also been rarely found in IMA (2.2%) [7]. IMA often displays multicentric, multilobar, and bilateral lung involvement [9], and IMA at the early stage is prone to be misdiagnosed as lobar pneumonia or other diffuse lung lesions. Meanwhile, IMA may be discovered accidentally at an early stage by computed tomography (CT), and in the case of small lesions in the peripheral lung, preoperative diagnosis is frequently difficult by bronchoscopy or transthoracic biopsy [7]. Hence, a definitive diagnosis and treatment strategy may be determined through an intraoperative frozen section diagnosis (FSD). However, besides the poor quality of the fresh frozen sample [10], IMA can be often misdiagnosed or overlooked due to its mild structural and cytological atypia [11,12].

Herein, we report a case of IMA misdiagnosed as focal organizing pneumonia by intraoperative FSD. However, simultaneously performed touch imprint cytology (TIC) revealed nuclear inclusion in epithelial cells with a mucinous background, suggesting the possibility of malignancy. If the result of the TIC had been taken seriously by pathologists, the misdiagnosis might have been avoided. This experience will underscore the efficacy of TIC in combination with FSD in the intraoperative diagnosis of suspected IMA. In this setting, mucoid background and nuclear inclusion on the TIC may suggest IMA, even if the FSD does not suggest malignancy.

## 2. Case Report

**Figure 1:**
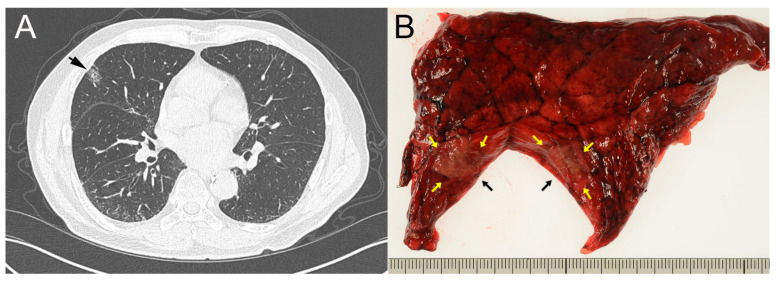
(**A**) A 15 mm-sized subsolid nodule was observed in the periphery of the upper lobe, S3, of the right lung (arrow). (**B**) Cut surface of the resected lung specimen. A grayish-brown nodule, 10 × 7 mm in size, was observed (arrows).

A 74-year-old Japanese man, under treatment of alcoholic liver cirrhosis, hepatocellular carcinoma and chronic renal failure, underwent a screening CT and a subsolid nodule with a diameter of 15 mm was incidentally pointed out in the right upper lobe of the lung (Figure 1A). He had no history of smoking. The tumor markers on admission were as follows: CEA 7.3 ng/mL (Reference, 0.0–5.0), CA19-9 < 2.0 U/mL (0.0–37.0), SCC 1.0 ng/mL (0.0–1.5), NSE 7.9 ng/mL (0.0–16.3), CYFR 1.3 ng/mL (0.0–3.5), Pro GRP 45.8 pg/mL (0.0–80.9).

**Figure 2:**
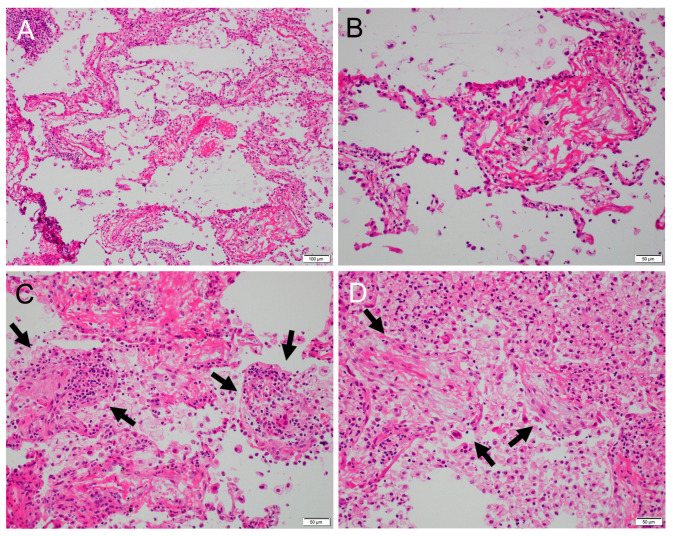
(**A**) Mild fibrotic thickening of alveolar septa, mimicking non-specific interstitial pneumonia (H&E, ×10). No sign of invasion was observed. (**B**) Mildly thickened alveolar septum was lined with a single layer of epithelium with mildly enlarged nuclei, which was interpreted as reactive type 2 pneumocyte (H&E, ×20). (**C**) Stromal fibrosis with moderate round cell infiltration was observed (arrows). (**D**) Process formation into the alveolar duct was observed (arrows), suggesting organizing pneumonia. The alveolar duct was filled with a lot of macrophages (H&E, ×20). Nuclear inclusions and mucus were not recognized in frozen section diagnosis.

**Figure 3:**
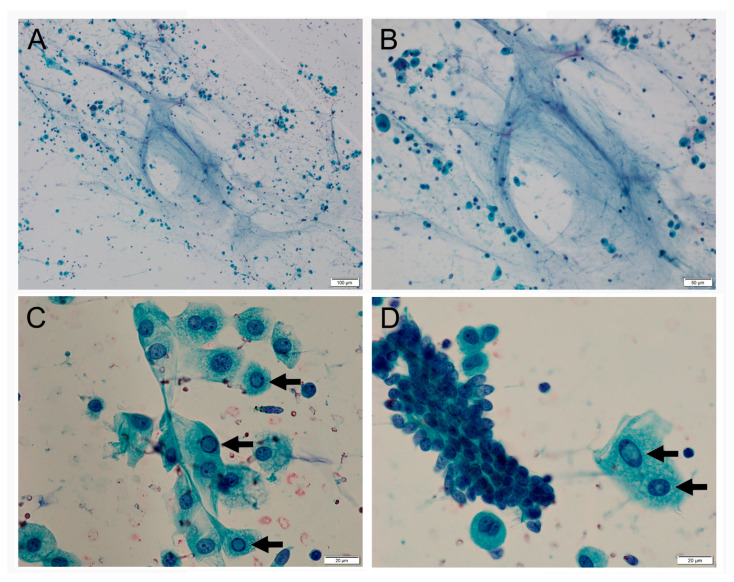
(**A**) Mucus and inflammatory cells in the background (Pap, ×10). (**B**) Mucus and inflammatory cells in the background (Pap, ×20). (**C**) Epithelial cells show mild nuclear enlargement with nuclear inclusion (arrows) and abundant cytoplasm. A dinucleated epithelial cell was observed (Pap, ×40). (**D**) Epithelial cells show mild nuclear enlargement with nuclear inclusion (arrows) and abundant cytoplasm. A dinucleated cell in this figure piece was considered to be a macrophage (Pap, ×40).

**Figure 4:**
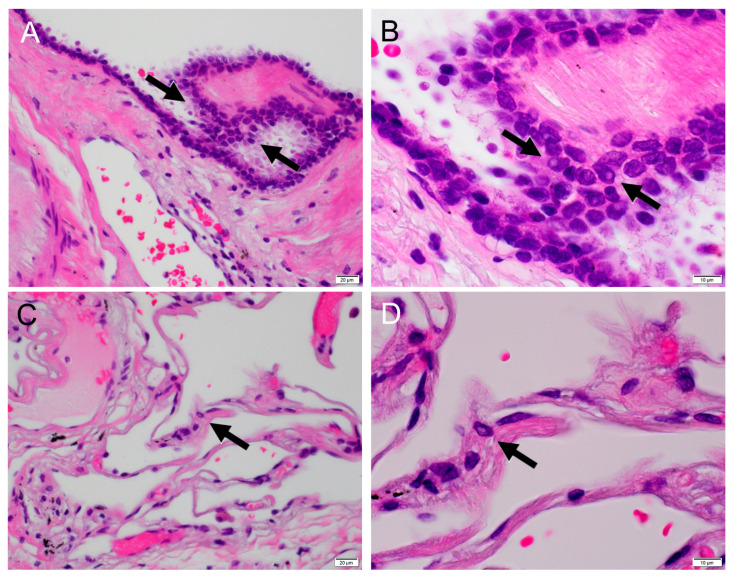
(**A**) Bronchiolar epithelium with nuclear inclusion (arrows) in normal lung tissue from an 83-year-old man (H&E, ×40). (**B**) Bronchiolar epithelium with nuclear inclusion (arrows) (H&E, ×100). (**C**) Alveolar epithelium with nuclear inclusion (arrows) in normal lung tissue from an 83-year-old man (H&E, ×40). The epithelial cell with the nuclear inclusion would likely to be a type 2 pneumocyte. (**D**) Alveolar epithelium with nuclear inclusion (arrows) (H&E, ×100).

Early lung cancer was suspected, and a wedge resection was performed by video-assisted thoracoscopic surgery (VATS). The wedge resection sample, 9 × 5 cm in size, was submitted and a grayish-brown tumor sized 10 × 7 mm was observed on the cut surface (Figure 1B). Intraoperative FSD revealed mildly thickened alveolar septa, lined with a single layer of cuboidal or low columnar epithelium with mildly enlarged nuclei, which pathologists interpreted as reactive type 2 pneumocyte (Figure 2A,B). Cytological and structural atypia was modest, and stromal invasion was not observed. In addition to stromal fibrosis, process formation surrounded by many foamy macrophages was observed in the alveolar duct (Figure 2C,D). Nuclear inclusion in the epithelial cells and mucinous background were not evident in the FSD. Simultaneously performed TIC revealed mucinous background with a lot of macrophages and nuclear inclusion in the epithelial cells (Figure 3A–D). However, two pathologists made a tentative diagnosis of focal organizing or non-specific interstitial pneumonia through discussion, because nuclear inclusion is occasionally observed in non-neoplastic lung tissue (Figure 4A–D). No atypical tissue was observed at the resection margin. As a result, an additional lobectomy and lymphadenectomy were not performed.

**Figure 5:**
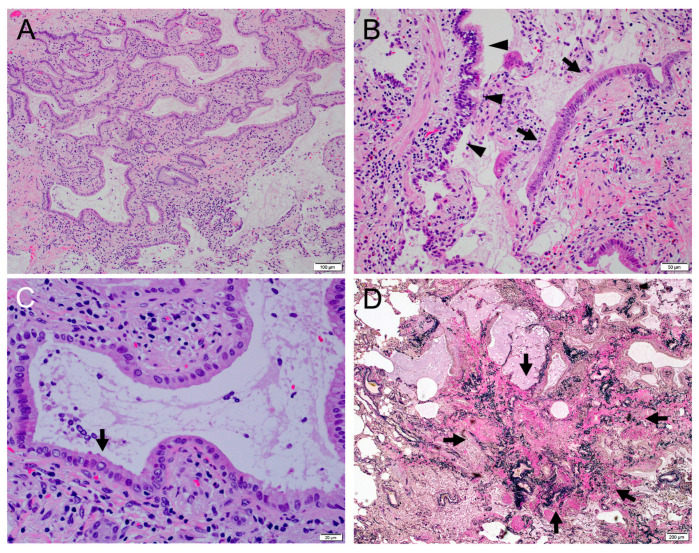
(**A**) Proliferation of high columnar epithelium with mucus production in a lepidic manner and thickening of the alveolar septum (H&E, ×10). (**B**) Normal bronchiolar epithelium (arrowhead) and neoplastic epithelium (arrow) (H&E, ×20) (**C**) A nuclear inclusion in cancer cells (arrow) (H&E, ×40) (**D**) A focus of stromal invasion, 3 mm in size. The disrupted elastic fiber network with increased collagen fiber can be observed (arrows) (EVG, ×10).

A permanent section diagnosis (PSD) revealed atypical high columnar epithelium with mucus production, which showed an abrupt transition from ciliated bronchiolar epithelium by hematoxylin and eosin (H&E) staining (Figure 5A,B). The nuclei were bright and slightly enlarged with swollen nucleoli. The nuclear edge was irregular. Intranuclear inclusions similar to those observed in TIC were also confirmed (Figure 5C). A 3 mm-sized microinvasion was observed by the Elastica van Gieson (EVG) staining (Figure 5D).

Immunohistochemical analysis revealed that the tumor cells were focally and weakly positive for CK20 and caudal-related homeobox 2 (CDX2) but were negative for TTF-1. The final pathological diagnosis was microinvasive IMA with negative resection margin.

The postoperative course was uneventful, and the patient was discharged on the 7th postoperative day. The patient has been disease-free for six months postoperatively.

## 3. Discussion

The aim of this case study was to highlight the efficacy of TIC in intraoperative diagnosis of IMA, with a special reference to nuclear inclusion. We hence discussed the limitation of FSD and the diagnostic utility of nuclear inclusion on TIC for the diagnosis of IMA.

The final diagnosis of this case was IMA by PSD. Morphology on H&E sections is usually sufficient for its diagnosis, but immunohistochemistry can play an ancillary role. IMA exhibits a distinct immunoprofile from that of INMA, which expresses CK7 and in about 75% cases TTF-1. In contrast, IMA expresses CK7 and focally coexpress CK20 and/or CDX2, but TTF-1 and napsin A are usually negative [2,13]. Our case was consistent with these findings. IMA must be differentiated from colloid adenocarcinoma, which exhibits a similar immunoprofile to IMA, such as positivity for CK7, CK20, and CDX2 [14]. Colloid adenocarcinoma consists of tumor cells morphologically similar to IMA but is characterized by abundant pools of extracellular mucin that distend alveolar spaces and destroy their walls [14]. More than 50% of the tumor area must be a colloid pattern [14]. In addition, IMA must be differentiated from metastatic adenocarcinoma from the pancreatobiliary system, gastrointestinal tract, and ovary, which also may exhibit similar morphologies and immunoprofiles with IMA [14,15,16]. In this study, the systemic work-up excluded metastatic lung cancer.

When a definitive diagnosis of the peripheral small nodules of the lung has not been reached preoperatively, intraoperative FSD is performed to confirm a diagnosis and guide the surgical strategy. Frozen sections are often of a low quality, making diagnosis difficult and requiring expertise, but accurate diagnosis is usually possible. Liu et al. reported that the total concordance rate between FSD and PSD was 84.4% among the 803 patients with clinical stage I peripheral lung adenocarcinoma who underwent sublobular resection [17]. However, IMA (formerly referred to as mucinous BAC) is sometimes overlooked or misdiagnosed because of the weak atypia. Marchevsky et al. reported that two of nine BAC less than 1.1 cm and one of five BAC measuring 1.1 to 1.5 cm were interpreted as alveolar hyperplasia or benign lesion by FSD [11]. Although tumor size was not described, Gupta et al. reported that seven and eleven BAC were misdiagnosed and deferred, respectively, among the 2405 frozen sections examined [12].

In this case study, the pathologists also misdiagnosed IMA with FSD because of weak cellular and structural atypia. Although cytotechnologists recognized and reported nuclear inclusions on intraoperative TIC that suggested malignancy, the pathologists considered them as reactive. As a result, the surgery was finished with a VATS wedge resection. However, the final diagnosis by PSD was IMA, in which nuclear inclusions were confirmed.

Cytology is commonly used to diagnose non-small cell lung cancer, but it is an inaccurate means of BAC diagnosis. The greatest issue is to distinguish between BAC and reactive bronchial/alveolar cells. Zaman et al. proposed predominance of two- and three-dimensional tissue fragments, tenacious intracytoplasmic connections between cells, intranuclear cytoplasmic inclusions, and paucity of multinucleated cellular forms as key cytological features favoring BAC [18]. Furthermore, they also reported significant differences between the mean of the nuclear area of benign reactive cells and that of the malignant cells [18]. The presence of mucus and predominance of mucinous cells may also favor BAC [19,20,21].

Nuclear inclusions are often observed in thyroid papillary carcinoma and breast cancer, and they are also used as one of the indicators of malignancy in cytology. Most of the intranuclear inclusions seen in primary lung adenocarcinoma are pseudointranuclear inclusions, where the nuclear membrane invaginates into the nucleus. Komatsu et al. reported that they appear in any of Clara cell type, goblet cell type, bronchial glandular cell type, type 2 pneumocyte-type and mixed-type lung cancer cells by electron microscopic findings and were recognized in 71% by cytology and 67% by histology [22]. In addition, nuclear inclusion was observed in 25 (100%) out of 25 BAC by Kasai et al. [23], 7 (30.4%) out of 23 BAC and 1 (8%) out of 12 invasive adenocarcinomas by Ohori et al. [19], and in 15 (68.2%) out of 22 papillary adenocarcinomas by Iyoda et al. [24]. Thus, nuclear inclusion is frequently observed in BAC, and it may be useful in picking BAC up. In fact, Raz et al. listed nuclear grooves and intranuclear inclusions as characteristic cytological findings in BAC [20]. On the other hand, there is a limitation of nuclear inclusion as an indicator of malignancy. Characteristic intranuclear inclusions are seen in viral infections such as the herpes virus, cytomegalovirus, adenovirus etc., and are used as indicators of viral infections [22]. Intranuclear inclusions also appear in sclerosing pneumocytoma [25], pulmonary fibrosis [26,27], and even in bronchial asthma due to air pollution, [28] and in normal lung tissue (Figure 4). Kawanami et al. reported nuclear inclusion in bronchiolar cuboidal and type 2 pneumocyte in 9 out of 11 patients with bronchial asthma associated with air pollution [28]. It should be noted that these benign diseases are all diffuse lung diseases, except for sclerosing pneumocytoma. Thus, nuclear inclusions can appear in inflammatory and benign contexts. According to our experience in clinical practice, nuclear inclusions in lung adenocarcinoma can be easily found, but those in non-neoplastic lung tissue cannot be found unless we search very closely. Although the exact frequency cannot be easily measured and there has been no literature, we assume that the frequency of nuclear inclusion would be much less in non-neoplastic lung tissue than in adenocarcinoma.

TIC is suitable for observing individual cells and mucinous background. Based on the above discussion, we think that TIC should be performed together with FSD in the intraoperative inspection of nodular lung lesions. When nuclear inclusions are recognized with mucinous background, BAC should not be excluded even if cellular and structural atypia are so weak.

## 4. Conclusions

Intraoperative inspection of peripheral small nodular lesions in the lung by FSD alone can result in misdiagnosis or the overlooking of IMA. TIC combined with FSD can be useful in the accurate diagnosis of IMA, because TIC is more suitable to observe nuclear inclusions and mucinous background than FSD.

## Data Availability

All data generated or analyzed in this study are included in this article.
